# Promoting carotenoid biosynthesis in the NUD(X23)

**DOI:** 10.1093/plcell/koae057

**Published:** 2024-02-20

**Authors:** Guy Levin

**Affiliations:** Assistant Features Editor, The Plant Cell, American Society of Plant Biologists; Faculty of Biology, Technion, Haifa 32000, Israel

Carotenoids are essential plant pigments, having roles in light harvesting, photoprotection, and antioxidant activity, among others. Carotenoid biosynthesis, or carotenogenesis, is a complex process facilitated by multiple proteins, starting with geranylgeranyl diphosphate synthase (GGPPS) and phytoene synthase (PSY) (see Fig.). GGPPS produces GGPP, a carotenoid precursor, and PSY catalyzes the condensation of 2 GGPP molecules to form phytoene, the first intermediate of carotenogenesis. In plants, the rate of carotenogenesis is limited mainly by PSY activity (Zhou et al. 2022), and, accordingly, PSY expression and activity are heavily regulated. Numerous transcription factors regulate *PSY* gene expression, while alternative splicing, protein-protein interactions, and other processes further modulate PSY activity (Liang and Li 2023). In Arabidopsis, one such protein-protein interaction is that of GGPPS11 with PSY, which promotes the channeling of GGPP to PSY (Ruiz-Sola et al. 2016). Interestingly, it was recently revealed that some members of the enzyme family NUcleoside Diphosphates linked to moiety-X hydrolases (NUDXs) can hydrolyze various metabolites, some of which are precursors of carotenoids, suggesting they may also influence carotenogenesis (Henry et al. 2018). However, the function of many NUDX family members and their potential roles in carotenogenesis remain unknown.

**Figure.**
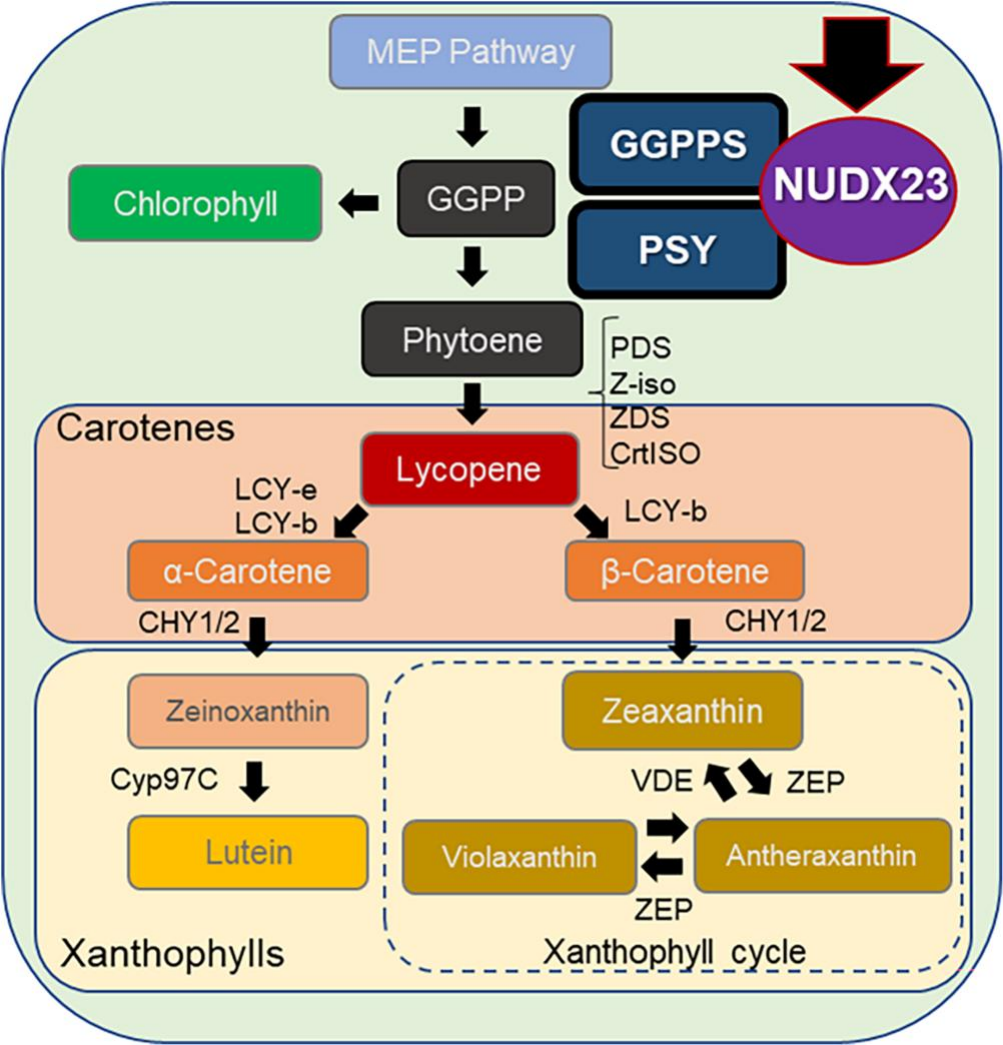
NUDX23 influences carotenoid biosynthesis by interacting with and enhancing the stability of the PSY-GGPPS complex, promoting phytoene generation. Further processing of phytoene in the carotenoid biosynthetic pathway provides essential carotenoids to the plant. Selected carotenoids and enzymes are shown.

In this issue of *The Plant Cell*, **Sombir Rao, Hongbo Cao, and colleagues** (Rao et al. 2024) identify NUDX23 as a crucial enzyme in the carotenoid biosynthesis pathway. Screening of a cauliflower cDNA library in an engineered *Escherichia coli* system that accumulates lycopene and expresses Arabidopsis *PSY* and *GGPPS11* genes identified 2 clones that decreased lycopene and encoded NUDX3. NUDX3 is a cytosolic protein known to dephosphorylate the carotenoid precursors isopentenyl pyrophosphate, dimethylallyl pyrophosphate, farnesyl pyrophosphate, and geranyl pyrophosphate to their respective monophosphate products (Henry et al. 2018), and this likely explains the decreased lycopene levels in the *E. coli* system. However, PSY and GGPPS were unexpectedly accumulated in the NUDX3 encoding clones, and the Nudix domain of NUDX3 was shown to directly interact with GGPPS and PSY. This has prompted an investigation to test if chloroplast-localized NUDXs influence carotenogenesis.

In Arabidopsis, NUDX14, NUDX19, NUDX23, NUDX26, and NUDX27 are targeted to the chloroplast. Expressing the individual cDNAs in the engineered bacterial system revealed that the cells expressing NUDX23 and NUDX27 contained elevated lycopene levels *and* elevated PSY, as well as GGPPS protein levels. NUDX23 is highly expressed in leaf tissue, and overexpressing NUDX23 in Arabidopsis enhanced the accumulation of carotenoids *and* PSY and GGPPS proteins. Interestingly, the transcript levels of carotenoid pathway genes were not elevated, implying that NUDX23 influences carotenogenesis post-translationally. Additional analysis showed that when protein synthesis is inhibited, Arabidopsis plants overexpressing NUDX23 maintain high levels of PSY and GGPPS, suggesting that NUDX23 affects their stability or turnover rate. Importantly, NUDX23 co-migrated with PSY and GGPPS in blue-native gel electrophoresis, indicating it is part of the PSY-GGPPS complex that channels GGPP to the carotenoid biosynthesis pathway (Ruiz-Sola et al. 2016). In *nudx23* knock-out Arabidopsis lines, PSY and GGPPS did not co-migrate, suggesting NUDX23 is crucial for assembling the PSY-GGPPS complex. Finally, quantification of carotenoids in Arabidopsis *nudx23* mutants confirmed NUDX23 is crucial for carotenoid accumulation in plants. Overall, these results reveal that NUDX23 promotes carotenogenesis by facilitating the formation of a large PSY-GGPPS-NUDX23 complex, where it stabilizes PSY and GGPPS (see Fig.).

In addition to their importance for plants, carotenoids provide major health benefits for humans, and enhancing carotenoid production in crops has been a long-standing goal for scientists (Paine et al. 2005). Here, the authors revealed that NUDX23 promotes carotenogenesis in plants through interaction with and stabilization of the PSY-GGPPS complex, which channels carotenoid precursors into the carotenoid biosynthetic pathway. This study provides important insights into the fine-tuning of carotenogenesis and offers a potential target, NUDX23, to enhance carotenoid production in crops.

## Dive Curated Terms

The following phenotypic, genotypic, and functional terms are of significance to the work described in this paper:

GGPP CHEBI: CHEBI:57533

CAROTENOID CHEBI: CHEBI:23044

PSY Gramene: AT5G17230
PSY Araport: AT5G17230

DMAPP CHEBI: CHEBI:16057

FPP CHEBI: CHEBI:17407

GPP CHEBI: CHEBI:17211

IPP CHEBI: CHEBI:128769

## References

[koae057-B1] Henry LK , Thomas ST, Widhalm JR, Lynch JH, Davis TC, Kessler SA, Bohlmann J, Noel JP, Dudareva N. Contribution of isopentenyl phosphate to plant terpenoid metabolism. Nat Plants. 2018:4(9):721–729. 10.1038/s41477-018-0220-z 30127411

[koae057-B2] Liang M-H , Li X-Y. Involvement of transcription factors and regulatory proteins in the regulation of carotenoid accumulation in plants and Algae. J Agric Food Chem. 2023:71(48):18660–18673. 10.1021/acs.jafc.3c05662 38053506

[koae057-B3] Paine JA , Shipton CA, Chaggar S, Howells RM, Kennedy MJ, Vernon G, Wright SY, Hinchliffe E, Adams JL, Silverstone AL, et al Improving the nutritional value of golden rice through increased pro-vitamin A content. Nat Biotechnol. 2005:23(4):482–487. 10.1038/nbt1082 15793573

[koae057-B4] Rao S , Cao H, O'Hanna FJ, Zhou X, Lui A, Wrightstone E, Fish T, Yang Y, Thannhauser T, Cheng L, et al Nudix hydrolase 23 post-translationally regulates carotenoid biosynthesis in plants. Plant Cell. 2024:36(5):1868–1891. 10.1093/plcell/koae030 PMC11653588 38299382

[koae057-B5] Ruiz-Sola MÁ , Coman D, Beck G, Barja MV, Colinas M, Graf A, Welsch R, Rütimann P, Bühlmann P, Bigler L, et al *Arabidopsis* GERANYLGERANYL DIPHOSPHATE SYNTHASE 11 is a hub isozyme required for the production of most photosynthesis-related isoprenoids. New Phytol. 2016:209(1):252–264. 10.1111/nph.13580 26224411

[koae057-B6] Zhou X , Rao S, Wrightstone E, Sun T, Lui ACW, Welsch R, Li L. Phytoene synthase: the key rate-limiting enzyme of carotenoid biosynthesis in plants. Front Plant Sci. 2022:13:884720. 10.3389/fpls.2022.884720 35498681 PMC9039723

